# Quantifying Two-Dimensional Filamentous and Invasive Growth Spatial Patterns in Yeast Colonies

**DOI:** 10.1371/journal.pcbi.1004070

**Published:** 2015-02-26

**Authors:** Benjamin J. Binder, Joanna F. Sundstrom, Jennifer M. Gardner, Vladimir Jiranek, Stephen G. Oliver

**Affiliations:** 1 School of Mathematical Sciences, University of Adelaide, Adelaide, South Australia, Australia; 2 School of Agriculture, Food and Wine, Waite Campus, University of Adelaide, Adelaide, South Australia, Australia; 3 Cambridge Systems Biology Centre and Department of Biochemistry, University of Cambridge, Cambridge, United Kingdom; Massachusetts Institute of Technology, United States of America

## Abstract

The top-view, two-dimensional spatial patterning of non-uniform growth in a *Saccharomyces cerevisiae* yeast colony is considered. Experimental images are processed to obtain data sets that provide spatial information on the cell-area that is occupied by the colony. A method is developed that allows for the analysis of the spatial distribution with three metrics. The growth of the colony is quantified in both the radial direction from the centre of the colony and in the angular direction in a prescribed outer region of the colony. It is shown that during the period of 100–200 hours from the start of the growth of the colony there is an increasing amount of non-uniform growth. The statistical framework outlined in this work provides a platform for comparative quantitative assays of strain-specific mechanisms, with potential implementation in inferencing algorithms used for parameter-rate estimation.

## Author Summary

In nutrient-depleted environments, it is commonly observed that strains of the yeast *Saccharomyces cerevisiae* forage by the mechanisms of filamentous and invasive growth. How do we quantify this spatial patterning of outward growth from a yeast colony? Previous studies have primarily relied on measuring the amount of growth, but do not take into account the spatial distribution of this highly non-uniform process. We fill this void by providing a statistical approach that enables the quantification of this important spatial information. This approach enables a more detailed mathematical analysis of the growth process and should allow the precise definition of mutant phenotypes, thus enabling a detailed analysis of the genetic control of morphogenesis.

## Introduction

**Figure 1:**
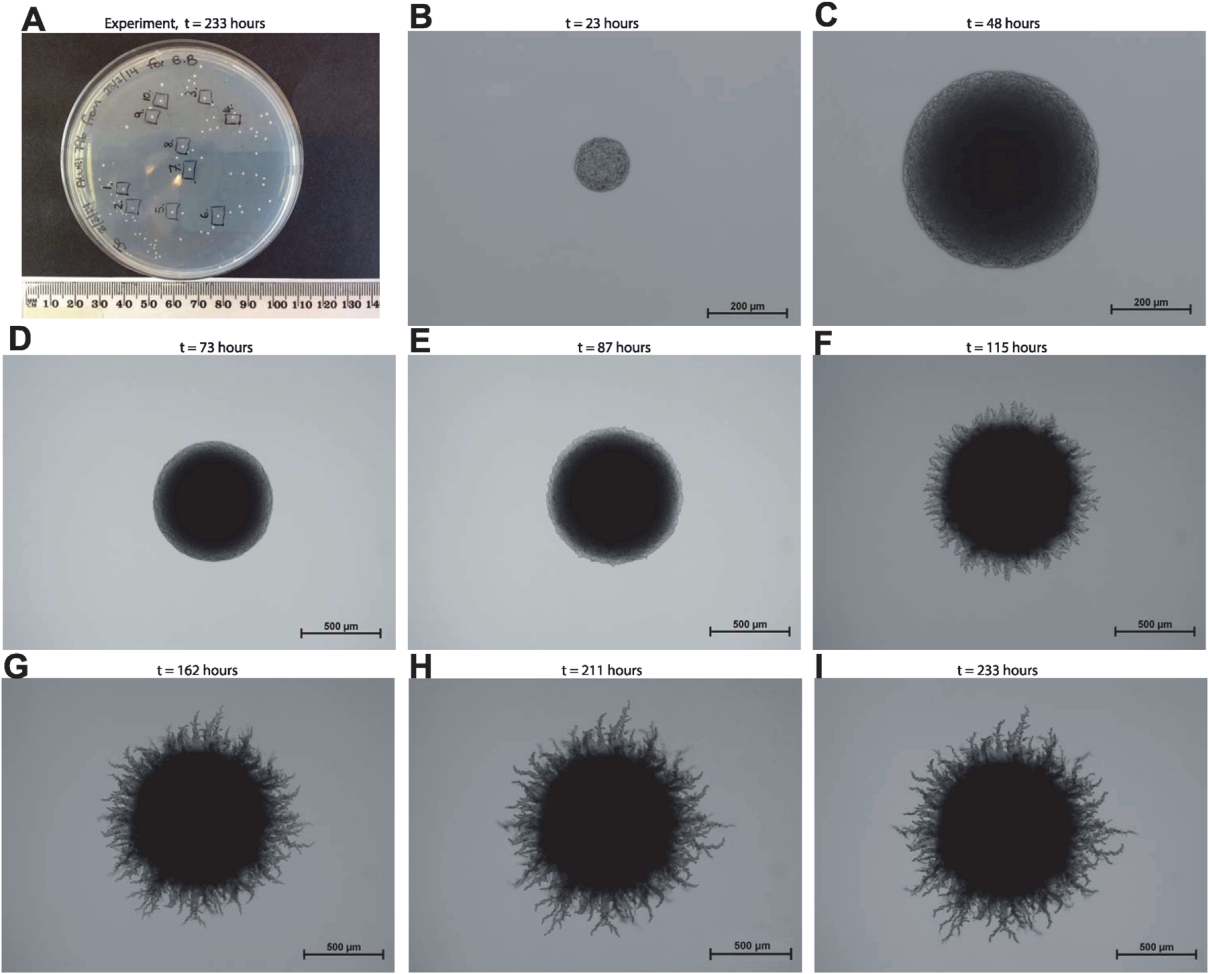
Filamentous growth of a yeast colony (*Saccharomyces cerevisiae*; AWRI 796 yeast strain with 50 *μ*M ammonium sulphate nutrient concentration). (a) Image of the experiment taken at 233 hours. A sample of 10 colonies was analysed. (b)–(i) Experimental images of colony number 5 taken at time *t* = {23, 48, 73, 87, 115, 162, 211, 233} hours.

Many strains of the bread, wine and ale yeast *Saccharomyces cerevisiae* are dimorphic, which means they are able to grow either by the budding of single cells or as multicellular filaments called pseudohyphae [Fig. 1]. These filaments differ from the true hyphae of most fungi and some yeasts (notably the human pathogen *Candida albicans*) in that they lack the elongated tubular structure of true hyphae and, instead, are made up of chains of unseparated cells. The transition between the budding and pseudohyphal modes of growth is triggered when *S. cerevisiae* is subjected to glucose or, especially, nitrogen deprivation or when the yeast is treated with fusel alcohols [1]. It is thought that the pseudohyphae perform an adaptive function, enabling non-motile *S. cerevisiae* to forage for new growth substrates in a nutritionally poor environment. This may have relevance to this yeast’s lifestyle in the wild, since both grape juice and oak sap are known to be nitrogen-poor [2, 3] and pseudohyphal growth may allow *S. cerevisiae* to adhere to or penetrate plant tissues and thereby gain access to new sources of nitrogen.

Much is known about the genetic control of pseudohyphal growth and quantitative assessments of the phenotype have been made in order to screen the library of *S. cerevisiae* single-gene deletion mutants for genes involved in filament formation [4]. These assessments have relied on commercial imaging software developed to measure the outgrowth of neurites from nerve cells [5]. In other comparative quantitative assays, both filamentous and invasive growth is measured by the relative volume and area of the colony [4, 6, 7]. While such software has proven its worth in these screens, it does not provide an adequate basis for the further mathematical analysis or modelling of filamentous growth in *S. cerevisiae*. This is because they neglect spatial information regarding the location or positioning of cell-area that is occupied by the colony. We address this deficiency, developing a methodology that allows us to formulate three spatial metrics to quantify the two-dimensional spatial distribution of occupied cell-area within the circular domain that is a yeast colony.

The three spatial metrics (*radial, angular, angular pair-correlation*) quantify the spatial patterning of a colony’s deviation from spatial homogeneity, or the complete spatial randomness (CSR) state [8–11]. This is similar to existing statistics used in the analysis of spatial point data sets including: quadrat methods [8–13], nearest-neighbour functions [8–10], *K*-functions [8–10] and pair-correlation functions [10, 14–20]. We analyse spatial data sets, that identify area within the spatial domain that is occupied by the cells of the colony, obtained from processing two-dimensional experimental images of yeast colonies [19, 20].

The analysis is carried out at length-scales where individual cells are visible in the images at early times [Figs. 1(b) and (c)], with the detailed spatial patterning of the entire colony being captured at later times [Figs. 1(d) and (i)]. This enables us to quantitatively distinguish between the patterning of surface filamentous growth and invasive growth into the medium, but not the mechanisms at the individual cell level for the filamentous and invasive growth processes. We show that the time course of colony development can be characterised with indicators for growth in the radial direction from the centre of the colony, growth in the angular direction in a prescribed outer annular region of the colony, and localised aggregation (angular direction) in a prescribed outer annular region of the colony. All three indicators show that there is an increasing amount of non-uniform growth during the period between 100 and 200 hours after the initiation of growth of each colony by a single cell.

Our statistic-based methodology has immediate utility in the detailed quantitation of filamentation phenotypes resulting from either mutations or exposure to chemicals. This will allow a detailed and quantitative assessment of morphological phenotypes in high-throughput assays of libraries of single-gene deletants (as in [4]). Moreover, the quantitative nature of our phenotypic analysis using these metrics particularly lends itself to the elucidation of the genetic interactions involved in yeast morphogenesis using double-gene deletants generated by the synthetic genetic array (SGA; [21]) approach. In addition, we believe, it provides a basis for a more detailed mathematical analysis of the processes and mechanisms involved in filamentous and invasive growth.

## Results

Data sets are extracted from black and white images [e.g. Fig. 4(a)] of yeast colonies in experiments similar to that shown in Fig. 1(a) (see Materials and Methods). Black pixels in the images represent cell-area that is occupied by the colony, within a circular domain, *x* ^2^ + *y* ^2^ ≤ *R* ^2^. The pixels (black or white) in these binary images define a two-dimensional square (outer) domain, consisting of an integer lattice with unit spacing. At discrete points in time, *t*, measured in hours, the lattice sites (*x*, *y*), with *x*, *y* ∈ [−*R*, *R*], are either occupied (black pixels) or unoccupied (white pixels) by the cell-area of the colony [19, 20]. This is denoted with a matrix

$$M(x,y)=\{\begin{array}{l} 0\;\text{if}\;x^{\text{2}}\;+\;y^{\text{2}}>R^{\text{2}},\\ 0\;\text{if}\;\text{site}\;(x,y)\;\text{is}\;\text{vacant}\;\text{and}\;x^{\text{2}}+y^{\text{2}}\leq R^{\text{2}},\\ 1\;\text{if}\;\text{site}\;(x,y)\;\text{is}\;\text{occupied}\;\text{and}\;x^{\text{2}}+y^{\text{2}}\leq R^{\text{2}}. \end{array}$$ (1)

The number of occupied lattice sites within the circular domain is

$$n=\sum_{x,y}M(x,y),$$ (2)

with corresponding mean-field density

$$\rho =\frac{n}{\pi R^{2}}.$$ (3)

The spatial positioning of the occupied sites on the lattice is analysed with three metrics.

### Spatial metrics

Three spatial metrics are formulated to quantify the spatial distribution of occupied sites within the circular domain [8–20]. The first metric is a scaled count of the number of occupied sites in annuli from the centre of the domain. The second is a scaled count of the number of occupied sites in circular segments of the domain. The third is a scaled count of the number of (acute) angles between all possible combinations of pairs of occupied sites in the domain; for example, a pair of occupied sites (1, 0) and (0, 1) have a pair angle of 90° between them. We refer to the three metrics as the radial, angular and angular pair-correlation metric, respectively. The radial and angular pair-correlation metrics are invariant to rotation of the domain, whilst the angular metric depends on the orientation of the domain.

Next, we consider the set of position vectors for the occupied sites within the circular domain

P={v(x,y)∣M(x,y)=1,x,y∈[−R,R]}. (4)

The subsets for the magnitudes and principle arguments of the position vectors, and angles between pairs of position vectors are given by

$$S_{r}(i)=\left\{v|\Delta_{r}(i-1)\leq \;|\;v\;|\;<\Delta_{r}i,v\in P\right\},\text{ for }\Delta_{r}=\frac{R}{L}\text{ and }i=1,\ldots ,L;$$ (5)

$$S_{\theta }(j)=\left\{v|-\pi +\Delta_{\theta }(j-1)\leq \arg(v)<-\pi +\Delta_{\theta }j,v\in P\right\},\text{ for }\Delta_{\theta }=\frac{2\pi }{M}\text{ and }j=1,\ldots ,M;$$ (6)

$$S_{\Theta }(k)=\left\{(v_{1},v_{2})|\Delta_{\Theta }(k-1)\leq \cos^{-1}\left(\frac{v_{1}\cdot v_{2}}{\left|v_{1}||v_{2}\right|}\right)<\Delta_{\Theta }k,v_{1},v_{2}\in P\right\},\text{ for }\Delta_{\Theta }=\frac{\pi }{N}\text{ and }k=1,\ldots ,N;$$ (7)

where *L*, *N*, and *M* are the number of equally spaced partitions on the corresponding intervals [0, *R*), [−π, π) and [0, π). The counts of the number of radial distances and arguments of the position vectors, and angles between pairs of position vectors are then

$$c_{r}(i)=|S_{r}|,\;c_{\theta }(j)=|S_{\theta }|\text{ and }c_{\Theta }(k)=|S_{\Theta }|.$$ (8)

To obtain the radial, angular and angular pair-correlation metrics, the counts are scaled or normalised with respect to circular domains that are populated uniformly at random, or are at the CSR state [8–11]. For the radial counts, the expected number of occupied sites in each bin or annulus is the area, $A_{i}=\pi \Delta_{r}^{2}(2i-1)$, multiplied by the mean-field density *ρ*. This gives the radial metric

$$F_{r}(i)=\frac{c_{r}(i)}{\pi \rho \Delta_{r}^{2}(2i-1)}.$$ (9)

A similar argument holds for the angular counts, except in this case the bins are circular segments, giving the angular metric

$$F_{\theta }(j)=\frac{c_{\theta }(j)}{\frac{1}{2}\rho \Delta_{\theta }R^{2}}.$$ (10)

The normalisation values for the pair angle counts is based upon the observation (not shown) that the counts fluctuate around a constant value for all pair angles. Intuitively, due to the periodic nature of the counts, this makes sense as there should be no bias at any particular pair angle in a randomly populated circular domain. We therefore expect the counts at any pair angle to be the number of all possible combinations of pairs, $\left(\begin{array}{c} n\\ 2 \end{array}\right)=n(n-1)/2=\rho \pi R^{2}(\rho \pi R^{2}-1)/2$, divided by the number of bins, *N* = π/Δ_ϴ_, which gives the angular pair-correlation metric

$$F_{\Theta }(k)=\frac{c_{\Theta }(k)}{\frac{1}{2}\rho \Delta_{\Theta }R^{2}(\pi \rho R^{2}-1)}.$$ (11)

When evaluating the angular pair correlation metric a subset of *n_s_* randomly selected occupied sites from the domain is analysed, as the number of calculations is computationally expensive, being of $O(n^{2})$. This gives an effective density of *ρ*_*s*_ = *n_s_*/π*R* ^2^ in the calculation of the angular pair-correlation metric (11).

**Figure 2:**
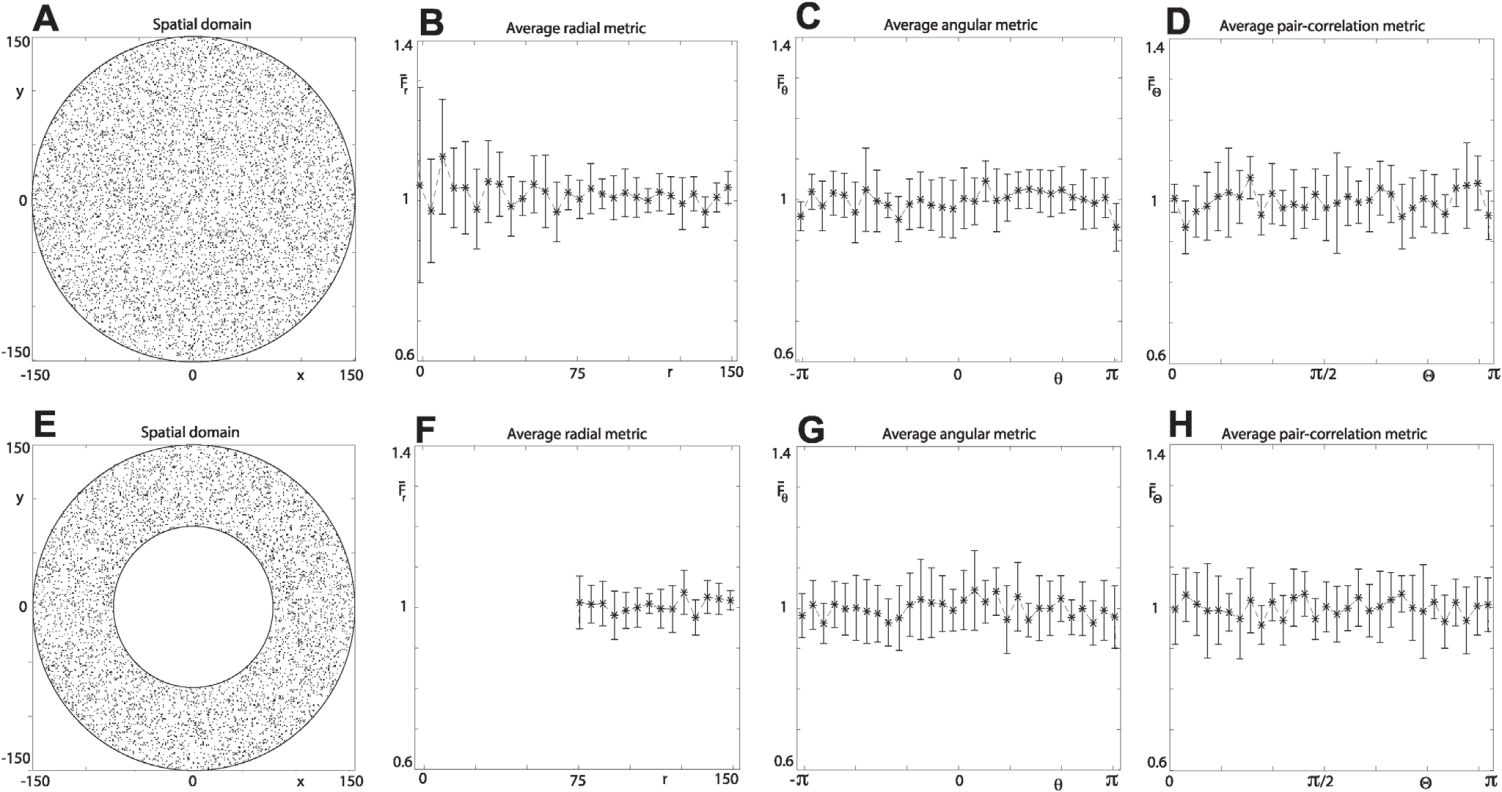
Spatial domains populated uniformly at random. Average values (markers) of the three metrics, with the standard error of the mean, from a sample of 10 domains populated uniformly at random. Circular domains *ρ* = 0.1, *R* = 150. Annulus domains *ρ*_*A*_ = 0.1, *R_A_* = 0.75, *ρ* = 0.076, *R* = 150. (a) Typical circular domain. (e) Typical annulus domain. (b) and (f) Average radial metric with *L* = 30 for the circular and annulus domains, respectively. (c) and (g) Average angular metric with *M* = 30 for the circular and annulus domains, respectively. (d) and (h) Average angular pair-correlation metric with *N* = 30 for the circular and annulus domains, respectively. In the calculation of the average angular pair-correlation metric *n_s_* = 100 occupied sites were selected at random from each domain in the sample.

To validate the analysis, we evaluate the three spatial metrics, given by Eqns. (9–11), for a sample of 10 circular domains populated uniformly at random [e.g. Fig. 2(a)], and plot their average values at the bin edges *r* = Δ_*r*_(*i*−1), *θ* = −π+Δ_*θ*_(*j*−1) and ϴ = Δ_ϴ_(*k*−1). As expected, the signals fluctuate around unity [Fig. 2(b)–(d)]. All three metrics can be implemented to analyse spatial patterns in annulus-shaped domains, with *R*_*A*_ ≤ ∣**v**∣ ≤ *R* [Fig. 2(e)–(h)]. For an annulus populated uniformly at random, there is no change in the formulation of the angular metric (10) or angular pair-correlation metric (11); however, the mean-field density *ρ* is replaced with $\rho_{A}=n/\pi (R^{2}-R_{A}^{2})$ in the formulation for the radial metric (9).

**Figure 3:**
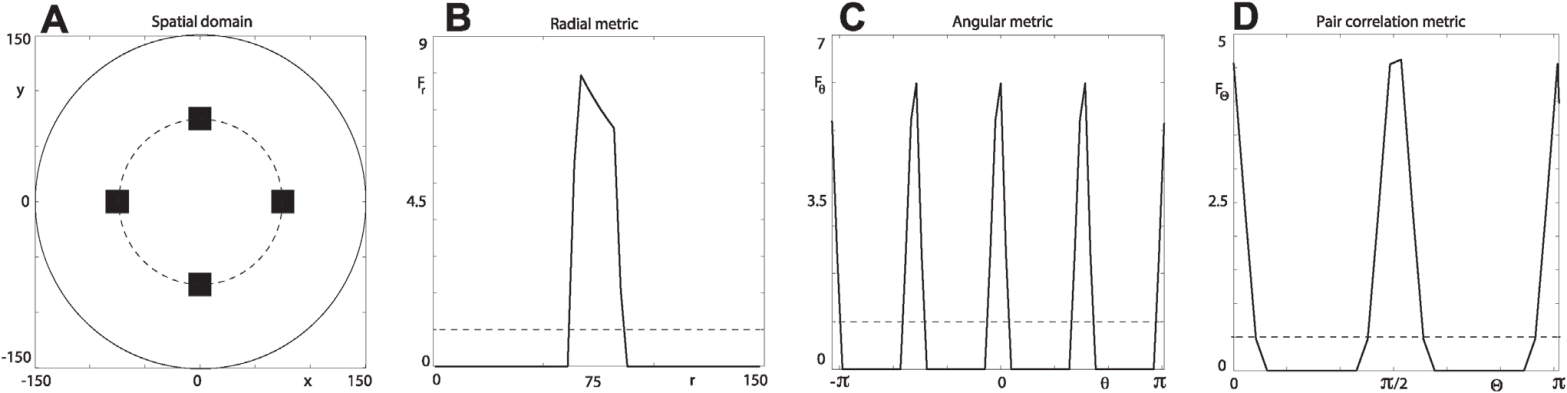
Regular spatial distribution. (a) Four 21 × 21 square shaped clusters centred on the circle (broken curve) with radius *R*/2 for a circular domain with *ρ* = 0.0250, *R* = 150. (b) Radial metric *F_r_* with *L* = 50 for (a). (c) Angular metric *F*_*θ*_ with *M* = 30 for (a). (d) Angular pair-correlation metric *F*_ϴ_ with *N* = 30 from *n_s_* = 1000 randomly selected occupied sites in the domain for (a). The three signals in (b)–(d) provide a quantitative way to characterise the spatial patterning.

Further understanding on interpreting the three spatial metrics is provided by analysing their signals for the regular patterning observed in Fig. 3(a). The pattern consists of four square-shaped clusters, each of length *l* = 21, equally spaced on a circle that is half the radius of the circular domain. We see that the signal for the radial metric in Fig. 3(b) has a maximum at a radial distance *r* ≈ *R*/2 = 75, indicating maximum aggregation. For radial distances *r* < *R*/2 − *l*/2 and $r>\sqrt{{\left(R/2+l/2\right)}^{2}+{\left(l/2\right)}^{2}}$ there is maximum segregation with *F_r_* = 0. When examining the signal for the angular metric [Fig. 3(c)], the three maxima at *θ* = {−π/2, 0, π/2} correspond to the three clusters with central position vector arguments *θ* = {−π/2, 0, π/2}. The two maxima at *θ* = {−π, π} correspond to the single cluster with central position vector argument *θ* = π. The results for the angular pair-correlation metric [Fig. 3(d)] have three maxima at ϴ = {0, π/2, π}. The first maximum corresponds to short-scale aggregation within each cluster. The second and third maxima correspond to intermediate and long-scale aggregation between pairs of clusters separated by the angles ϴ = π/2 and ϴ = π.

### Experimental images

Samples of 10 colonies are analysed with the three spatial metrics [e.g. Fig. 1(a)]. We first analyse the time evolution of surface filamentous growth for a single colony [AWRI 796 yeast strain with 50 *μ*M ammonium sulphate nutrient concentration, Fig. 1(b)–(i)], before considering the average spatial-temporal patterning of filamentous growth for the entire sample of 10 colonies. Average values of the metrics are then compared with samples for washed colonies, a higher nutrient concentration (500 *μ*M) and a different strain of yeast (AWRI R2).

**Figure 4:**
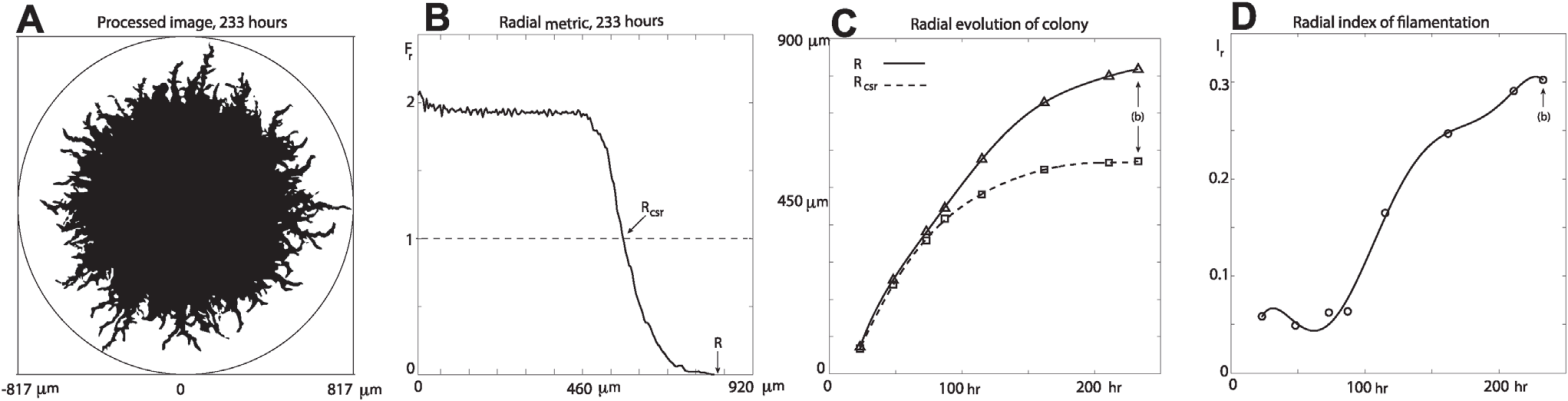
Radial analysis of the time evolution of colony number 5. The radial distances are reported in microns. (a) Image processing for Fig. 1(i). (b) Radial metric for (a), Δ_*r*_ = 4.6, *R* = 817.3, *R*_csr_ = 570.4. (c) Evolution of the maximum radial distance *R* (triangular markers) and complete spatial randomness radial distance *R*_csr_ (square markers) for the colony. The data points correspond to the times *t* = {23, 48, 73, 87, 115, 162, 211, 233} in hours. (d) Evolution of the radial index (circle markers) for the colony. A sextic polynomial (curve) is fitted to each data set in the panels (c) and (d).

We begin the analysis with the radial metric shown in Fig. 4(b) for the processed image Fig. 4(a) of colony number 5 at 233 hours [Fig. 1(i)]. The approximately constant value of the metric’s signal up to a radial distance of around 460 microns corresponds to the region of the colony that is fully occupied. At these distances the signal’s value is greater than unity and the spatial domain is aggregated or under-dispersed [10, 11, 15, 18], which means that the probability of finding an occupied site is more likely than that for the CSR state. At the radial distance *R*_csr_ ≈ 570 microns the signal intersects with unity, indicating that at this distance the probability of finding an occupied site is as equally likely as the CSR state. We note that this *R*_csr_ value may vary depending on the resolution in the experimental images. For radial distances greater than 570 microns the signal is less than unity, indicating segregation or over-dispersion [10, 11, 15, 18], where the probability of finding an occupied site is less likely than the CSR state.

Results in Fig. 4(c) illustrate the time evolution of the colony’s maximum radial distance *R* and CSR radial distance *R*_csr_. We see that for early times the two radial distances are more or less indistinguishable, deviating from each at around 100 hours. This indicates that the colony’s growth is approximately uniform up to this time, and the deviation at later times is a quantifiable measure of the colony’s filamentation in the radial direction. This measure of filamentation can be summarised by defining a radial index

$$I_{\text{r}}=1-\frac{R_{\text{csr}}}{R},\text{ for }0\leq I_{\text{r}}\leq 1.$$ (12)

Values (markers) of the radial index in Fig. 4(d) show that there is a period of 100–200 hours with increasing filamentous growth of the colony. The radial index is (spatially) non-dimensional and therefore yields a quantitative measure that may be used to compare radial filamentous growth between two or more colonies.

**Figure 5:**
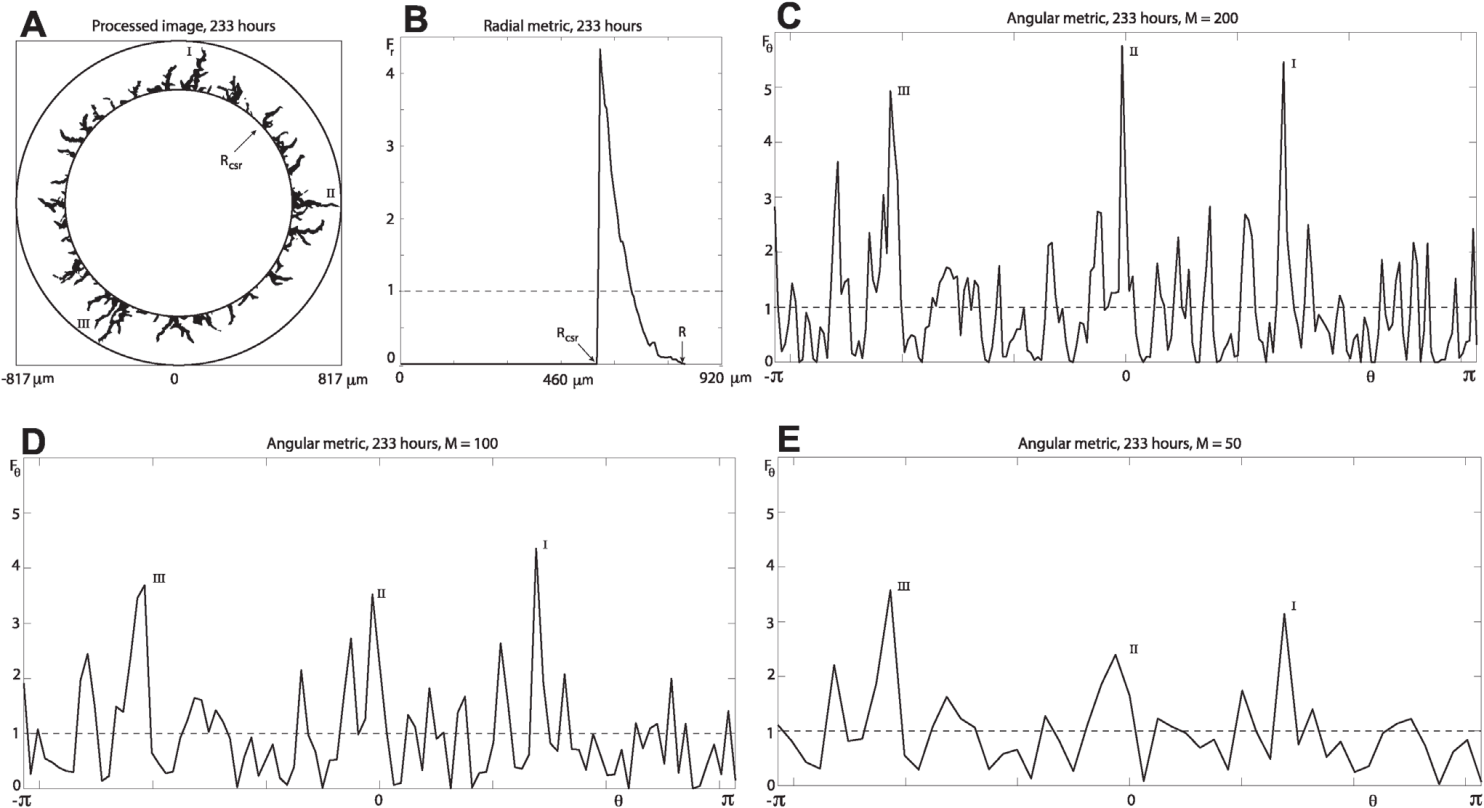
Radial and angular metric for an annular region of colony number 5 at 233 hours. The annular region is given by *R*_csr_ < ∣**v**∣ < *R*, with *R* = 817.3, *R*_csr_ = 570.4. (a) Image processing for Fig. 1(i). (b) Radial metric for (a), Δ_*r*_ = 4.6. (c)–(e) Angular metric for (a), with *M* = {200, 100, 50}.

The radial CSR distance and maximum radial distance naturally lead to the definition of an annular region of the domain, *R*_csr_ ≤ ∣**v**∣ ≤ *R*, dominated by filamentous growth [Fig. 5(a)]. This gives a region of the domain to analyse with the angular metric and angular pair-correlation metric that would otherwise be dominated by the fully occupied region of the colony as in the case of the entire circular domain [Fig. 4(a)]. The signals for the radial, angular and pair-correlation metrics are shown in Fig. 5(b), Fig. 5(c)–(e) and Figs. 7(a)–(b), respectively, for growth within the annular region of Fig. 5(a).

At radial distances greater than *R*_csr_, we observe that the radial signals for the entire circular domain [Fig. 4(b)] and annular region of the domain [Fig. 5(b)] are qualitatively similar. In other words, there is little additional information gained about the radial growth of the colony by further examination of the radial metric in the annular region. However, the maxima (above unity) in the angular metric signal (for three bin sizes) in Figs. 5(c)–(e) indicate angles of *θ* where the spatial domain is aggregated. The three largest maxima of each of the three signals are identified (approximately) on the processed experimental image Fig. 5(a). For increasing values of the bin width there is a reduction in the number of maxima in the angular signals [Fig. 5(c)–(e)], effectively averaging the data.

The results show that the angular metric can detect the orientation of filaments, which could prove useful in quantifying directionally biased growth in experiments with non-homogenous environments. For example, assessing whether there is any directional bias in growth towards a nutrient source. Of course, such assessments would rely on the angular metric being able to measure directionally biased growth relative to random fluctuations in a control experiment with no nutrient source.

**Figure 6:**
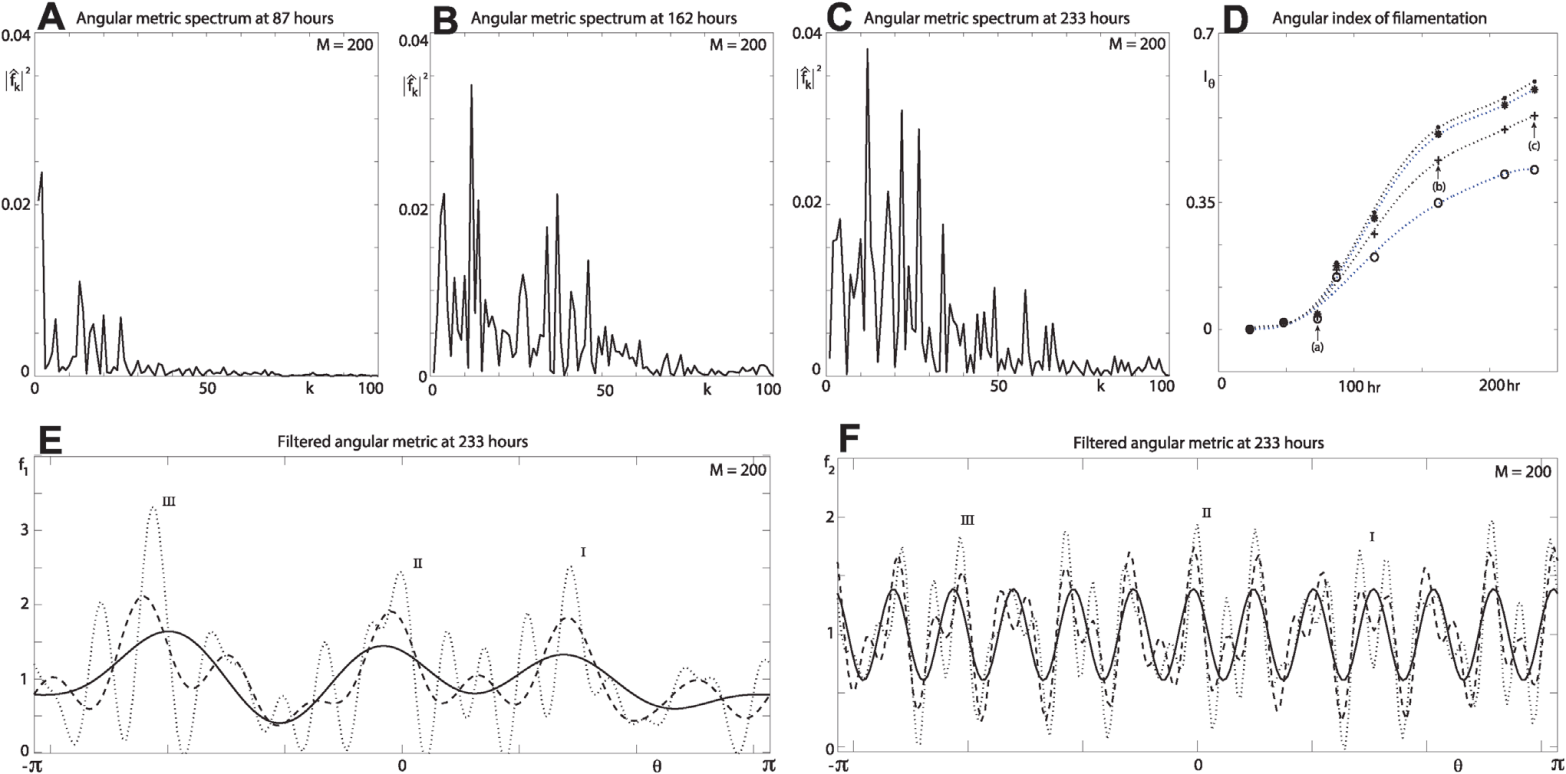
Spectral analysis of the angular metric, colony number 5. (a)–(c) Spectrum at *t* = {87, 162, 233} hours for wave numbers *k* = 1, …, *M*/2, *M* = 200. The spectrum in panel (c) is for angular metric signal in Fig. 5(c). (d) Evolution of the angular index (markers) at *t* = {23, 48, 73, 87, 115, 162, 211, 233}. A sextic polynomial (curve) is fitted to each data set. The broken curves (bottom to top) are for *M* = {100, 200, 500, 1000}. (e) and (f) Filtered signal for the angular metric in Fig. 5(c), at time *t* = 233 hours, *M* = 200. (e) Filtered signal *f*_1_. The solid, dashed and dotted curves are for *α* = {5, 10, 20}. (f) Filtered signal *f*_2_. The solid, dashed and dotted curves are for *β* = {0.035, 0.03, 0.025}.

The angular metric is further examined by taking its discrete Fourier transform [22]

$${\hat{f}}_{k}=\frac{1}{Mc_{k}}\sum_{j=0}^{M-1}F_{\theta }(j)e^{-\text{i}kx_{j}},\;k=0,\pm 1,\ldots ,\pm M/2,$$ (13)

where *x_j_* = −π + 2π*j*/*M*, *j* = 0, …, *M* − 1; *c_k_* = 2 for *k* = ±*M*/2, and *c_k_* = 1 for *k* ≠ ±*M*/2. The spectrum of the angular metric is plotted for increasing values of time in Figs. 6(a)–(c), and the evolution is summarised by defining the angular index of filamentation,

$$I_{\theta }=\sum_{k=1}^{M/2}|{\hat{f}}_{k}{|}^{2},$$ (14)

which is shown for four different bin sizes in Fig. 6(d). We see that, for early times, the angular index has approximately the same constant value, indicating uniform growth in the angular direction. This is followed by a period of increasing values of the index, indicating increasing filamentous growth, consistent with the results for the radial metric. We also observe that, as the bin size decreases, more modes are available to capture higher frequencies, resulting in higher values of the angular index at any given point in time during the period of filamentous growth [Fig. 6(d)]. The index (14) provides the means to compare angular filamentous growth between different yeast colonies, and filtering the angular signal gives an additional or alternative measure for such comparisons.

We filter the angular signal by considering two subsets of the modes,

$$K_{1}=\left\{k|\;|k|\;<\alpha ,k\in [-M/2,M/2]\right\}$$ (15)

and

$$K_{2}=\left\{k||{\hat{f}}_{k}{|}^{2}>\beta ,k\in [-M/2,M/2]\right\}.$$ (16)

The corresponding filtered signals are then given by the inverse discrete Fourier transform

$$f_{m}=\sum_{k\in K_{m}}{\hat{f}}_{k}e^{\text{i}k\theta },\;-\pi \leq \theta <\pi ,\text{ for }m=1,2.$$ (17)

The first filter *f*_1_ removes high frequencies from the signal and, as the number of modes decreases, the three maxima are filtered from the angular signal [Fig. 6(e)]. The second filter *f*_2_ identifies dominant modes in the signal, which can determine a characteristic wavelength [Fig. 6(f)] of the pseudohyphal filaments observed in Fig. 5(a).

**Figure 7:**
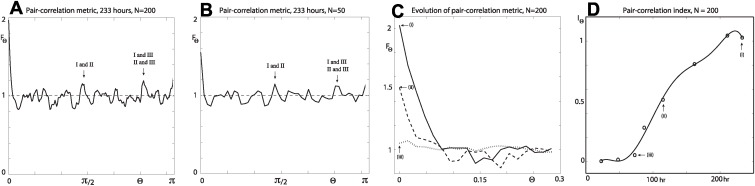
Angular pair-correlation metric for the annular filamentous region of colony number 5. The results are for *n_s_* = 1000 randomly selected occupied sites in the domain. (a)–(b) Pair-correlation signal for Fig. 5(a) at *t* = 233 hours, with *N* = {200, 50}. (c) Evolution of the pair-correlation signal. The dotted, broken and solid curves are for *t* = {73, 115, 233} hours, with *N* = 200. (d) Pair-correlation index of aggregation (markers) at *t* = {23, 48, 73, 87, 115, 162, 211, 233}, with *N* = 200. A sextic polynomial (curve) is fitted to the data set.

Presented in Fig. 7 is analysis of the annular region of the colony with the angular pair-correlation metric. Increasing the bin width [Figs. 7(a)–(b)] effectively averages the data, similar to that seen for angular metric’s results [Figs. 5(c)–(e)]. The maximum in the signal at the pair angle ϴ = 0 corresponds to localised aggregation, with pairs of position vectors belonging to the same filament. Maximum localised segregation occurs at the first minimum in the signal (below unity), quantifying the (average) angle separating the (more or less) distinct filaments. Remaining maxima for moderate and higher values of pair angles correspond to position vector pairs coming from two (more or less) distinct filaments [see Figs. 5(a) and Figs. 7(a)–(b)].

A measure of localised aggregation in the angular direction, as time evolves, is given by defining the pair-correlation index of aggregation as

$$I_{\Theta }=F_{\Theta }(1)-1,$$ (18)

which is plotted (markers) in Fig. 7(d). Similar to both the radial and angular index, the pair correlation index indicates a period of increasing localised aggregation between 100–200 hours.

**Figure 8:**
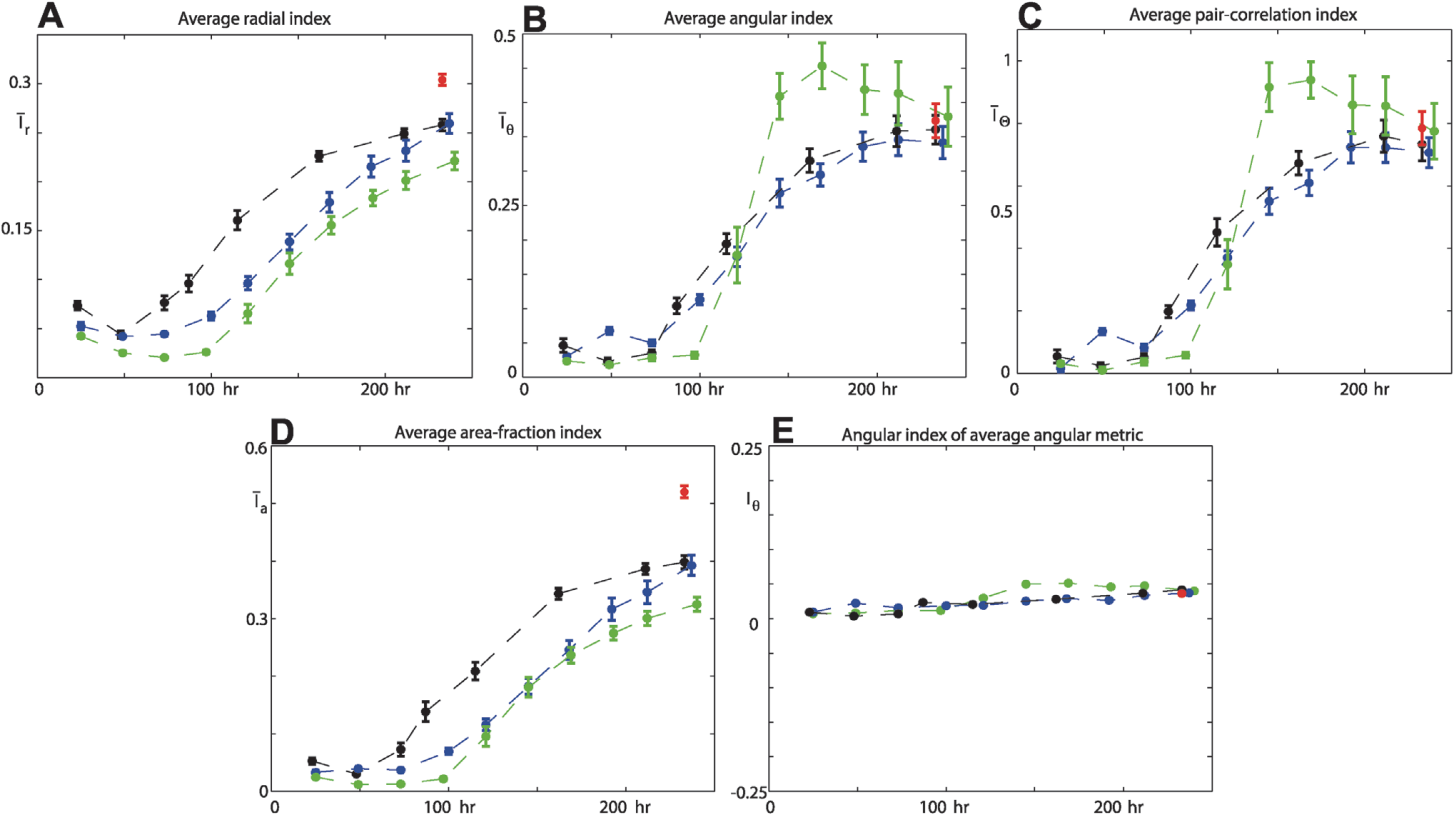
Spatial analysis for samples of 10 colonies. The black markers are the average indices, with the standard error of the mean, for filamentous growth of the AWRI-796 strain with 50 *μ*M ammonium sulphate nutrient concentration. The red markers (t = 233 hours) are the average indices for invasive growth of the AWRI-796 strain with 50 *μ*M ammonium sulphate. The green markers are the average indices for filamentous growth of the AWRI-796 strain with 500 *μ*M ammonium sulphate. The blue markers are the average indices for filamentous growth of the AWRI-R2 strain with 50 *μ*M ammonium sulphate. (a) Average radial index, Δ_*r*_ = 4.6. (b) Average angular index, *M* = 200. (c) Average pair-correlation index, *N* = 200. (d) Average area-fraction index. (e) Angular index of the average angular metric, *M* = 200.

Having seen how the three indices, given by Eqns. (12, 14, 18), can describe the evolution of spatial patterning for a single colony, we now examine the variability in filamentous growth by computing the average of the three indices [black markers in Figs. 8(a)–(c)] from a sample of 10 colonies. The 10 colonies used in the analysis are indicated on the image of the experiment in Fig. 1(a). The (black) error bars in Figs. 8(a)–(c) show a moderate amount of variability about the average values of the indices, suggesting that the spatial patterning of the AWRI-796 yeast strain, with 50 *μ*M ammonium sulphate nutrient concentration, can be characterised in a reliable way by the three average indices. This demonstrates potential for comparison with average indices of washed colonies [e.g. AWRI-796 with 50 *μ*M ammonium sulphate, red markers in Figs. 8(a)–(c)], variable nutrient concentrations [e.g. AWRI-796 with 500 *μ*M ammonium sulphate, green markers in Figs. 8(a)–(c)] and other yeast strains [e.g. AWRI-R2 with 50 *μ*M ammonium sulphate, blue markers in Figs. 8(a)–(c)]. The results indicate that the spatiotemporal growth processes can be quantitatively distinguished using our metrics.

To conclude this section, we consider the existing method of estimating the area-fraction occupied by the colony during the growth process [4, 6, 7], which is simply the mean field density, *ρ*, in Eqn. (3). An area-fraction index can then be defined as

$$I_{\text{a}}=1-\rho ,\text{ for }0\leq I_{\text{a}}\leq 1.$$ (19)

We see that trends in the average values, for both filamentous and invasive growth, of the area-fraction index in Fig. 8(d) are similar to those for the radial index in Fig. 8(a). However, the area-fraction index tells us nothing about the spatial patterning, other than that there is generally a decrease in density as time proceeds. For example, we could populate circular domains uniformly at random [e.g. Fig. 2(a)] to precisely the same densities found in each of the samples at the time points in the experiments to obtain exactly the same average area-fraction index values in Fig. 8(d). This further highlights that our three metrics provide important additional information on the spatial pattering of non-uniform growth of a yeast colony.

## Discussion

Quantifying the spatial patterning of a yeast colony is important in the comparision of strain-specific abilities to forage in nutrient-depleted environments and identifying genes involved in filamentous and invasive growth processes. Prior comparative analyses of filamentous and invasive growth have relied on commercial software to estimate the relative area of the colonies [4, 6, 7]. However, these quantitative assays neglect positional information within the spatial domain that is occupied by the cells of the colony. We have addressed this lack in utility of spatial information by developing three metrics that measure a colony’s radial and angular pattern formation. Our framework provides an alternative way to characterise and quantify filamentous and invasive growth, with analysis of the colony’s spatial distribution complementing existing methods.

To demonstrate the potential of the methodology we examined the spatial distribution of a single colony’s filamentous growth. The radial metric provided a measure of filamentous growth in the radial direction from the centre of the colony, and enabled us to define an outer annular region of the colony to analyse with the angular metric and angular pair-correlation metric. We showed that the growth can be summarised with three indices, all indicating a period of increasing non-uniform growth between 100–200 hours, and when considering a sample of 10 colonies we found a moderate variability about the average values of the three indices. Although the three indices provide a straightforward measure for use in comparative assays, the metrics upon which they are based may prove more informative in characterising both filamentous and invasive growth. For example, the angular metric could quantify the orientation of directed filamentous growth towards a nutrient source, whereas the corresponding angular index only provides a measure of the overall filamentous growth in the angular direction.

While both the angular metric and angular pair-correlation metric quantify the spatial patterning in the angular direction, they offer distinctive advantages over each other, depending on how they are implemented. For the example of directed growth towards a nutrient source, the angular pair-correlation metric will not identify the orientation of the source, but the signal (and pair-correlation index) is likely to have a higher value at short length-scales than that found in control experiments. This is because the angular pair-correlation metric is invariant to rotation of the spatial domain. In contrast, it is inappropriate to use the angular metric to obtain an average signal from a sample of colonies all growing in a homogenous nutrient environment. Due to the angular metric’s dependence on orientation, the averaged signal approaches unity as the number of samples increases, which is reflected in the angular index of the average angular metric in Fig. 8(e). In this case, the angular pair-correlation metric should be used to quantify the average angular spatial patterning, potentially being able to identify average strain-specific characteristics such as filament size and the multiple scales of filament separation.

In addition to characterising the spatial distribution of filamentous and invasive growth in future work on comparative quantitative assays, the three metrics could be implemented in an inferencing algorithm, such as approximate Bayesian computation [23–26], to estimate rates for the mechanisms of filamentous and invasive growth. This relies on developing a model of the filamentous and invasive growth processes, such as agent-based models, which have been successfully used to simulate other cell biological processes [13, 19, 20]; and finding summary metrics that are close to *sufficient*—effectively summarising *all* the spatial information in the experimental data. We believe that a combination of the three spatial metrics will yield a summary statistic suitable for the purpose of inferencing, and investigation of this is left to future research.

Finally, we mention that the methodology developed in this work can be used to describe the spatial patterning in circular domains of other physical systems. For example, the distribution of (i) locations of yeast colonies in experiments similar to Fig. 1(a), (ii) mammalian cells in experiments and simulations of circular barrier assays [27, 28], and (iii) tracer fluid particles in experiments and simulations of batch mixer devices, where the vat containing the fluid is a cylinder [29, 30].

## Materials and Methods

Single cells were used to initiate the growth of individual yeast colonies. Two-dimensional images of the time course of colony development were image processed to obtain black and white images. These binary images provided the data for the statistical analysis of the spatial patterning of the colonies filamentous and invasive growth. Described below are the experimental methods and imaging techniques.

### Media, strains and microbiological techniques

Yeast Nitrogen Base (YNB) medium was prepared using dehydrated culture media as per the manufacturer’s instructions (BD; Cat No. 233520) with the addition of glucose (2%) and ammonium sulphate (50 *μ*M or 500 *μ*M) as the sole nitrogen source, or as described previously for ‘carbon base for nitrate assimilation test’ [31] with the following modifications; glucose (2%), inositol (11.7 mg/L), and ammonium sulphate (50 *μ*M or 500 *μ*M). For 50 *μ*M ammonium sulphate YNB, pH was adjusted to 6.0 using sodium hydroxide. Media was subsequently filter sterilised. Synthetic Low Ammonium Dextrose (SLAD) medium was prepared based on SLAHD medium [32] with the omission of histidine and at the two ammonium sulphate concentrations (50 *μ*M and 500 *μ*M). Bacto agar (4%) (Becton Dickinson) was washed twice in ultrapure water and autoclaved to sterilise. Equal volumes of 2 × YNB (50 *μ*M or 500 *μ*M ammonium sulphate) and 4% molten Bacto agar were combined and 20 mL aliquots poured into standard 90 mm polystyrene Petri dishes. AWRI 796 and AWRI R2, diploid prototrophic wine strains of *S. cerevisiae*, were used in the experiments. The yeast were cultured from glycerol stocks in 2 mL YNB (50 *μ*M ammonium sulphate) for two days, at 28°C, with agitation. Dilutions, calculated to contain between 50 to 100 cells, were then spread on to SLAD agar and the plates incubated at 28°C to yield single colonies. For yeast grown on 500 *μ*M ammonium sulphate SLAD, cells were collected from late exponential phase cultures.

### Microscopic imaging

Yeast colonies were imaged successively over time [e.g. Fig. 1]. Bright field images were viewed at 40 × magnification using a Nikon Eclipse 50i microscope and imaged using a Digital Sight DS-2MBWc camera and NIS-Elements F 3.0 imaging software (Nikon), at time points measured in hours. Following initial imaging for filamentous growth of the AWRI 796 (50 *μ*M) strain, colonies were washed using a stream of ultrapure water, thus removing the cells from the surface enabling subsequent imaging of the invasive growth into the SLAD medium.

### Image processing

Experimental images were converted into black and white images [e.g. Fig. 4(a)] using customised software developed with Matlab’s image processing toolbox [19, 20]. Black pixels in the images represent area that is occupied by the cells of the colony. The centre of the occupied area of the colony (i.e. centre of mass) was found, along with the maximum radial distance *R* that any part of the colony (black pixel) was from this point. The centre and radial distance *R* were then used to define the circular domain of the colony.

The data corresponding to the unwashed and washed AWRI 796 (50 *μ*M) images, AWRI 796 (500 *μ*M) images, and AWRI R2 (50 *μ*M) images are stored in four Matlab files. Each file contains a four-dimensional array with binary entries: an entry of one indicates the location of unit size area that is occupied by the colony. The first and second dimensions in the array corresponds to the *x* and *y* Cartesian coordinates in the images, the third dimension corresponds to the time point and the fourth dimension indicates the sample number. The four data files can be found in the (S1–S4 Datasets).

## Supporting Information

S1 Dataset Data for AWRI 796 with 50 μM ammonium sulphate images, AWRI 796 50.mat (MAT)

S2 Dataset Data for AWRI 796 with 50 μM ammonium sulphate washed images, AWRI 796 50 washed.mat (MAT)

S3 Dataset Data for AWRI 796 with 500 μM ammonium sulphate images, AWRI 796 500.mat (MAT)

S4 Dataset Data for AWRI R2 with 50 μM ammonium sulphate images, AWRI R2 50.mat (MAT)
